# Rapid Quantification of Methamphetamine: Using Attenuated Total Reflectance Fourier Transform Infrared Spectroscopy (ATR-FTIR) and Chemometrics

**DOI:** 10.1371/journal.pone.0069609

**Published:** 2013-07-30

**Authors:** Juanita Hughes, Godwin Ayoko, Simon Collett, Gary Golding

**Affiliations:** 1 Discipline of Chemistry, Faculty of Science & Technology, Queensland University of Technology, Brisbane, Queensland, Australia; 2 Chemical Analysis, Queensland Health Forensic and Scientific Service, Brisbane, Queensland, Australia; University of Quebect at Trois-Rivieres, Canada

## Abstract

In Australia and increasingly worldwide, methamphetamine is one of the most commonly seized drugs analysed by forensic chemists. The current well-established GC/MS methods used to identify and quantify methamphetamine are lengthy, expensive processes, but often rapid analysis is requested by undercover police leading to an interest in developing this new analytical technique. Ninety six illicit drug seizures containing methamphetamine (0.1%–78.6%) were analysed using Fourier Transform Infrared Spectroscopy with an Attenuated Total Reflectance attachment and Chemometrics. Two Partial Least Squares models were developed, one using the principal Infrared Spectroscopy peaks of methamphetamine and the other a Hierarchical Partial Least Squares model. Both of these models were refined to choose the variables that were most closely associated with the methamphetamine % vector. Both of the models were excellent, with the principal peaks in the Partial Least Squares model having Root Mean Square Error of Prediction 3.8, R^2^ 0.9779 and lower limit of quantification 7% methamphetamine. The Hierarchical Partial Least Squares model had lower limit of quantification 0.3% methamphetamine, Root Mean Square Error of Prediction 5.2 and R^2^ 0.9637. Such models offer rapid and effective methods for screening illicit drug samples to determine the percentage of methamphetamine they contain.

## Introduction

Worldwide, amphetamine-type stimulants (ATS), which include methamphetamine (MA), are ranked as the second most commonly used drug after cannabis. Up to 53 million people, i.e. 1.2% of the world population are estimated to have used an ATS in 2010. During the years 1998–2010 the seizures of ATS more than trebled and the seizure growth rates were far greater than those of the plant derived drugs (i.e. heroin, cocaine and cannabis). MA was the most prevalent ATS seized worldwide in 2010 with its seizure rates more than double that of two years earlier. [1].

The most common drug submitted for forensic analysis to Queensland Health Forensic and Scientific Services (QHFSS) is MA, accounting for approximately 40% of all submissions. This prevalence of one particular drug being presented for analysis to the scientists in the Illicit Drug section has led to an interest in developing a quick method of identifying and quantifying this illicit drug.

The current identification and quantification methods are time consuming and labour intensive processes with current turnaround times of more than a month from the time of submission. Police involved in undercover operations often require results within 24 hours which can lead to disruption of other analytical work. The streamlining of the committal process as part of the Moynihan reforms has lead to the police and prosecutors requesting the results of analysis earlier in the proceedings. [2] If the police can get indicative results of analysis within hours or one to two days then guilty pleas may be entered during the committal process eliminating much of the need for the current time consuming methods, and saving police, lawyers and court time, resulting in the saving of a significant amount of taxpayers’ money.

Fourier Transform Infrared Spectroscopy (FTIR) is a well established analytical technique for organic molecules, with the mid-IR region (4000 cm^−1^ to 400 cm^−1^) being rich in information about the structure of the functional groups within the analyte. FTIR can be used quantitatively, as the energy absorbed at a particular wavelength is in proportion to the number of bonds absorbing the associated quanta of energy, so with larger concentrations of analyte more of the energy will be absorbed.

The attenuated total reflectance (ATR) attachment for FTIR, allows direct measurement of the sample with minimal preparation and the potential to recover the sample, if required. ATR-FTIR is a reflectance method with the incident infrared radiation, reflecting off the attachment's crystal, penetrating into the sample then, reflecting back to the crystal.

The principal way that FTIR is used currently in the analysis of illicit drugs is through the use of spectral libraries to match the spectra of known compounds to the unknown (often a mixture). This technique is commonly used in identifying illicit drugs, precursors and other chemicals related to the process. [3], [4], [5] However, no robust methods have been published, to date, for the use of FTIR in the quantitative analysis of illicit drugs.

While ATR-FTIR has not been used in illicit drug quantification, ATR-FTIR with Chemometrics has been used to develop models within the pharmaceutical industry e.g. to quantify alterants, [6] and for the simultaneous quantification of multiple products for in-line quality control in manufacture. [7] So if working models can be developed for pharmaceutical drugs, there is a good potential that models for illicit drugs can also be developed. Goh's (2008) research was a proof of concept study. Though the number of genuine samples he used was limited, his work showed that ATR-FTIR was a promising technique for the in-field quantification of illicit drugs. [3].

The development of a method using ATR-FTIR and Chemometrics for rapid quantitative analysis of MA is the subject of this report. A successful method for this analysis would have not only national, but international implications, as it could be applied anywhere and the technique could be extended to other illicit drugs; and, with the use of available portable ATR-FTIR spectrometers and a laptop, could be applied for on-site analysis.

## Materials and Methods

### 1) Samples

The 96 samples used were subsamples of illicit drug seizures containing MA analysed by the Illicit Drug section of Forensic Chemistry at QHFSS. The subsamples were set aside by the scientists analysing the seizures after being homogenized. The MA % concentration of the samples was supplied by the QHFSS Illicit Drug section. This was determined by a National Association of Testing Authorities, Australia (NATA) approved, proprietary in-house method, using Ultra Performance Liquid Chromatography with Ultraviolet detection (UPLC-UV). In the samples analysed the concentration of MA ranged from 0.1% to 78.6%.

### 2) Total Attenuated Reflection Fourier Transform Infrared Spectroscopy

Triplicate spectra were acquired using a Thermo Scientific Nicolet™ 8700 Research FTIR Spectrometer with a single bounce diamond crystal ATR Smart iTR™ accessory which has a 1.5 mm active sample area, 2 µm penetration at 1000 cm^−1^ and ZnSe focusing optics. The resolution was approximately 2 cm^−1^ and 16 scans were accumulated. The IR spectra were recorded from 4000 cm^−1–^400 cm^−1^; however, the region from 650 cm^−1–^400 cm^−1^ was ignored because the ZnSe focusing optics have a lower wavelength limit cut-off of 650 cm^−1^.

### 3) Chemometric Analysis

#### a) Data collection

The spectral data for each sample was collected as a.CSV (comma-separated value ASCII) file. The data from the.CSV file opened within Excel format, and the triplicate spectral data was manually transferred to an Excel 2007 spreadsheet, which was designed to automatically perform data pre-treatment as explained below.

A spectral match was performed for each object using the inbuilt program, and the first several spectral matches and their percentages were recorded. As data about the other components within each sample was unavailable, this was used to approximate the cutting agent (with similar results grouped, e.g. powdered milk and propriety infant formulas = milk). When the concentration of methamphetamine in the sample was above 30%, it dominated the spectral matches; so it was difficult to determine the cutting agent, in any of these higher concentration methamphetamine samples.

#### b) Pre-treatment methods

**Figure 1:**
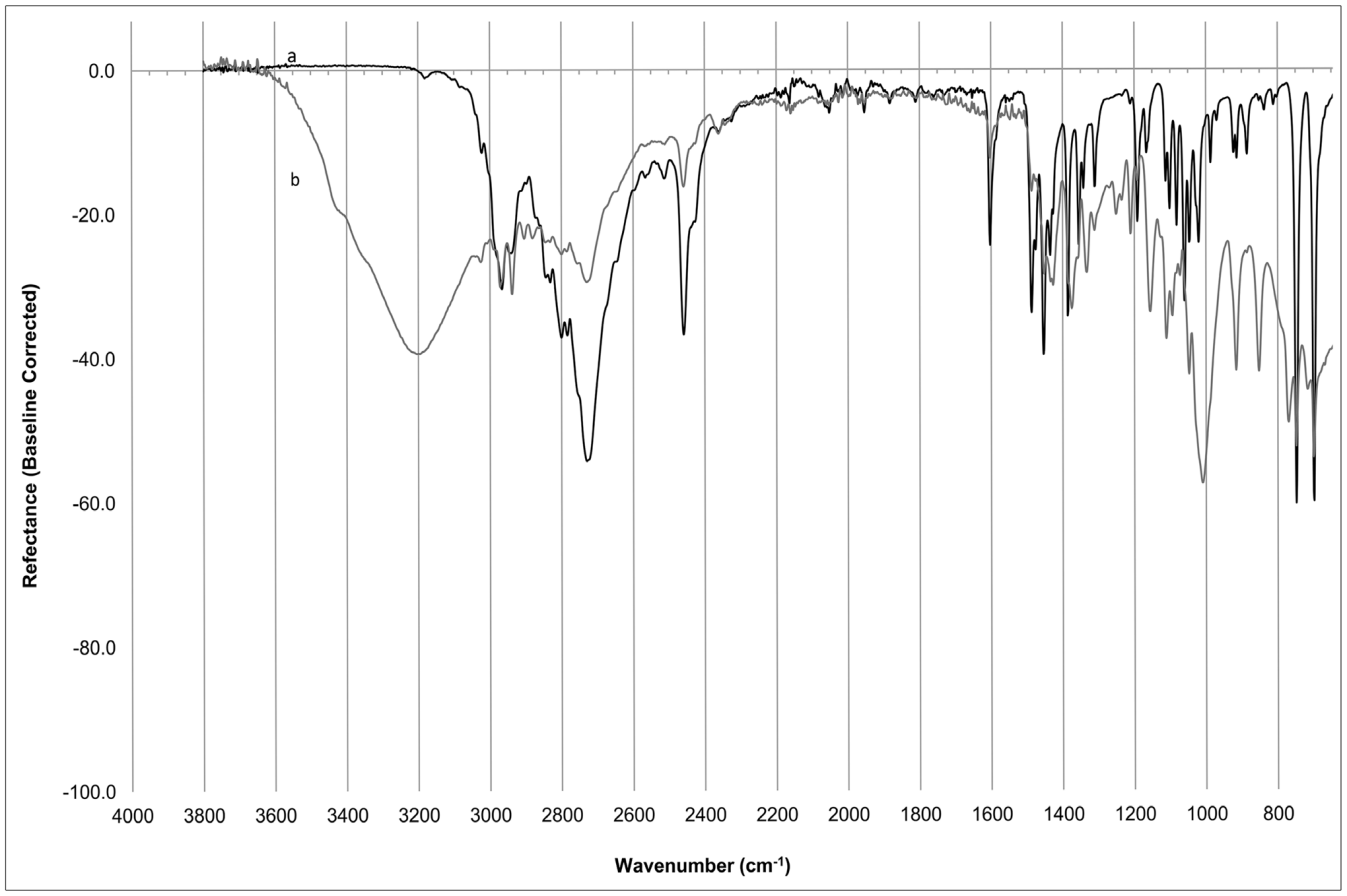
Typical ATR-FTIR spectra of Methamphetamine, a) High concentration {78.6%} b) Low concentration {10.3% cut with MSM (Methylsulphonylmethane)}.

The data for each of the triplicate spectra was baseline corrected using the featureless 3951 cm^−1^ region, see figure 1, and normalized. [3] The baseline correction was needed as the baselines varied during the analysis.

An Excel table was designed to do the above pre-processing; average the absorbance from the triplicate spectra; and to collect the results for the principal peaks of methamphetamine (determined by using a published ATR-FTIR Spectrum of methamphetamine); [5] as well as the hierarchical PCA (HPCA) data, as outlined below. The data was transferred manually to the appropriate tables for Chemometric analyses.

#### c) Partial Least Squares (PLS) Analysis using MA major peaks

The data was divided into training and test sets. The test set was partially (∼ 55%) from results when there were a large number of samples in one seizure. In this case, the samples were chosen in a way to as far as possible have similar samples in each of the training and testing groups. But if a unique sample was present, it was placed in the training group. The remaining objects in the test set (∼ 45%) were obtained later from totally unrelated samples.

Preliminary principal component analysis (PCA) was performed on the MA Major Peaks data. The software used for the Chemometrics analyses was SIMCA P+10 from Umetrics AB, Sweden. Prior to performing the preliminary PCA, the r^2^ value for each major peaks versus the supplied methamphetamine percentage was calculated using the RSQ function in Excel 2007. Excel’s RSQ function returns the square of r (the Pearson product moment correlation coefficient) where r is defined as:
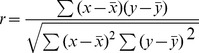



A high value of r^2^ (e.g. ≥0.85) at a particular major peak shows that the absorbance of the samples at that wavelength is highly correlated with the concentration of methamphetamine in the samples. The Major Peaks PCA was refined to select only those peaks that had an r^2^ value ≥0.85. These peaks were at the wavelengths (1387 cm^−^1, 1455 cm^−1^, 1487 cm^−1^, 1604 cm^−1^, 2460 cm^−1^, 2723 cm^−1^ and 2966 cm^−1^).

The major peak variables were used to perform a PLS analysis with the supplied methamphetamine percentage values used as the y variable. The method was refined by using only the variables with r^2^ values ≥0.85 as described above. Successive models were tried with various combinations of these variables until the combination with the highest R^2^ values, and lowest Root Mean Square Error of Estimation (training set) and Prediction (test set),RMSEE and RMSEP, values were obtained.

#### d) Hierarchical PLS Analysis (HPLS)

Janné et al [8] demonstrated hierarchical PLS is a useful data pre-treatment method for calibration. They showed that this technique can produce a model that can better predict lower values in the Y variable and achieve the best possible correlation between the X block and Y block variables. As a secondary PLS method, PLS analysis was performed on the significant PCs from a hierarchical PCA model (HPCA).

For the HPCA model, the spectra were divided into 10 blocks encompassing the regions 650 cm^−1^ to 1900 cm^−1^ and 2400 cm^−1^ to 3650 cm^−1^, see figure 1. The region between 1900 cm^−1^ and 2300 cm^−1^ was ignored because the Diamond crystal in the ATR attachment itself absorbs in this region. 2300 cm^−1^ to 2400 cm^−1^ was further eliminated as carbon dioxide has a major peak in this region. None of the spectra had any peaks from 3650 cm^−1^ to 4000 cm^−1^ so this region was also ignored. Each block covered a range of 250 cm^−1^, and contained 25 data points each being the average of 5 consecutive data points. PCA was performed on each block and a maximum of 3 significant PCs were recorded. These significant PCs from the initial PCA were used as the variables to perform a subsequent PCA, i.e. the HPCA.

**Figure 2:**
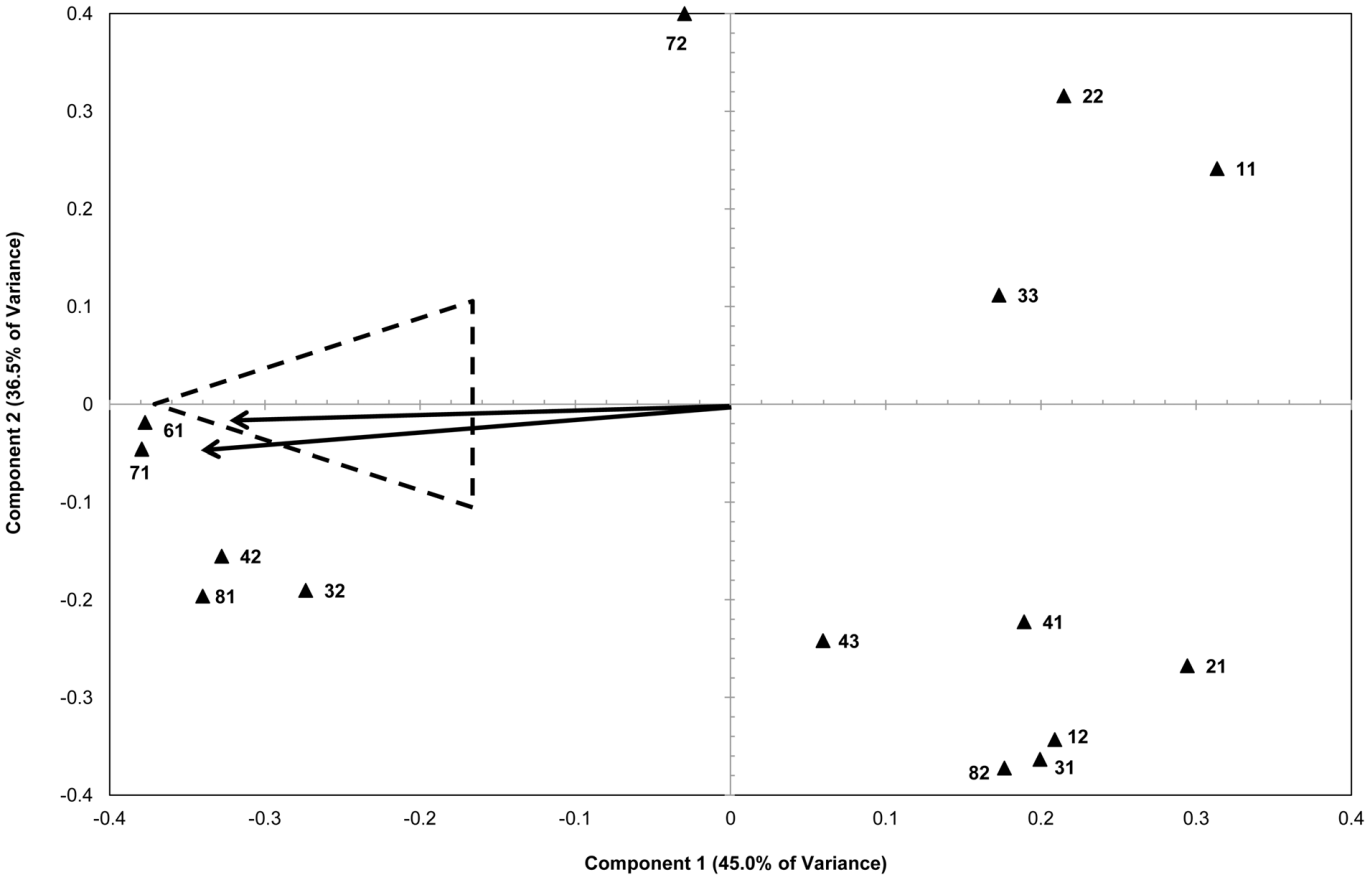
HPCA loadings plot: component 1 versus component 2. **(Dotted triangle shows Methamphetamine concentration direction in scores plot).**

The data for the HPLS analysis was separated as evenly as possible into training and test set. The 2 sets were tested for equivalence by checking that for each of the MA % ranges (<1, 1–10, 10–20 … >70), the number of objects within both the training and test sets were roughly equal; and that both sets contained even numbers of objects with the same cutting agents. The HPLS analysis was refined using the variable vectors clustering around and therefore roughly correlated to the high MA % objects, see figure 2 dotted area.

Both methods of analysis were refined by removing outlier objects to further improve the models. Objects which had variance between the known and predicted value of MA% of more than twice the value of the final RMSEE and RMSEP in the training and test set respectively were determined to be outliers.

#### e) Validation of models

The PLS models above were validated by cross validation, variable importance, external validation and response permutation. The cross validation was performed using R^2^Y (cum) and Q^2^ (cum) values produced by the SIMCA P-10 software. Sun, [9] states that accumulated values of Q^2^ (cum) >0.3 are statistically significant, >0.5 are good and >0.9 indicate that the model is excellent. Variable importance was compared with the level indicated by Sun, [9] i.e. level of the significance of importance  = 0.5. External validation was performed at a 95% confidence level, using the R^2^ value and RMSEP.

Response Permutation (20 permutations) was performed using the Validate Model function in the SIMCA P-10 software. The results were compared against the values given by Eriksson et al, [10] R^2^ below 0.3–0.4 and Q^2^ below 0.05, which are the limits they found by experience where the model is not over-fitted or over-predicted, indicating the model is not from randomly ordered Y data and therefore indicating the model validity.

## Results and Discussion

### 1) PLS Analysis Using MA Major Peaks

The final MA Major Peaks PLS Model had a RMSEE  = 3.5 and RMSEP  = 3.9. It had 3 significant components explaining 100% of the variance in the X space, cumulative R^2^Y  = 97.2% and cumulative Q^2^ = 96.7%. According to Sun, [9] accumulated values of Q^2^>0.9 indicate that the model is excellent. This model also shows a close cross validation with only 0.5% difference between cumulative R^2^Y and cumulative Q^2^ values.

The validated model Response Permutation % MA intercepts were R^2^ =  −0.0601, Q^2^ =  −0.317, well below R^2^ = 0.3–0.4, and Q^2^ = 0.05, the values reported by Eriksson et al [10] as the maximum values for a valid model. The 4 variables used in this model (1604 cm^−1^, 2460 cm^−1^, 2723 cm^−1^ and 2966 cm^−1^), each had variable importance (>0.98) well above 0.5 the level of significance indicated by Sun [9] indicating they are all highly relevant in explaining the model. They also had minimal difference in importance between the variables (0.047) that indicates they all have very similar relevance to the model. The highest values of variable importance belonged to the peaks at 2460 cm^−1^ and 2723 cm^−1^ which also had the highest RSQ values in the preliminary EXCEL analysis, see figure 1.

**Figure 3:**
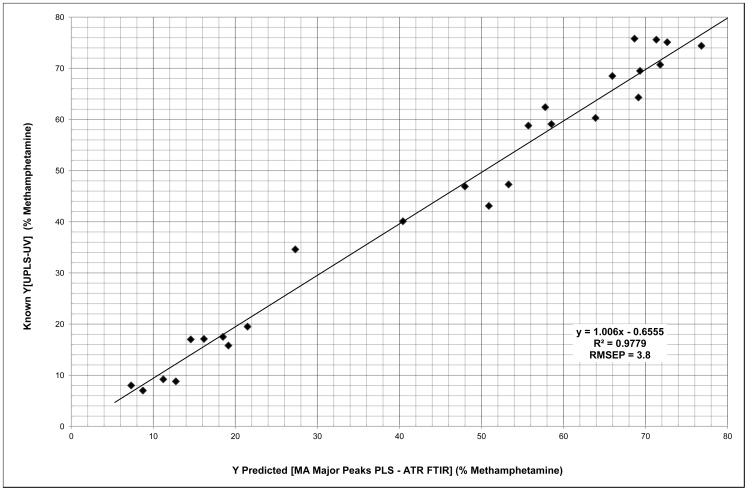
Methamphetamine Major Peaks PLS Regression.

With the Q^2^ value within the excellent range, close cross validation, response permutation significantly below the maximum values allowed, and all the variables used highly significant with similar relevance to the model; as well as the regression line of the training set with RMSEE of 3.5, coupled with the R^2^ value of 0.9779 and RMSEP of 3.8 for the test set indicates that this is a very good model, see figure 3.

The MA Major Peak PLS test set contains values from 7% up to 78.6% indicating it will predict methamphetamine concentrations approaching 80.3%, the theoretical maximum of the hydrochloride salt form. Considering that most methamphetamine is produced in ‘backyard’ laboratories, it is unlikely that a sample of 80.3% would be encountered. However, as 7% methamphetamine is the lowest concentration that has been tested in this model, we cannot safely project below this value. However, a promising fact is that the lowest concentrations within the model were well within the RMSEP value. The model may well be valid below this point though we cannot tell with the current set of objects.

### 2) Hierarchical PLS Analysis

**Figure 4:**
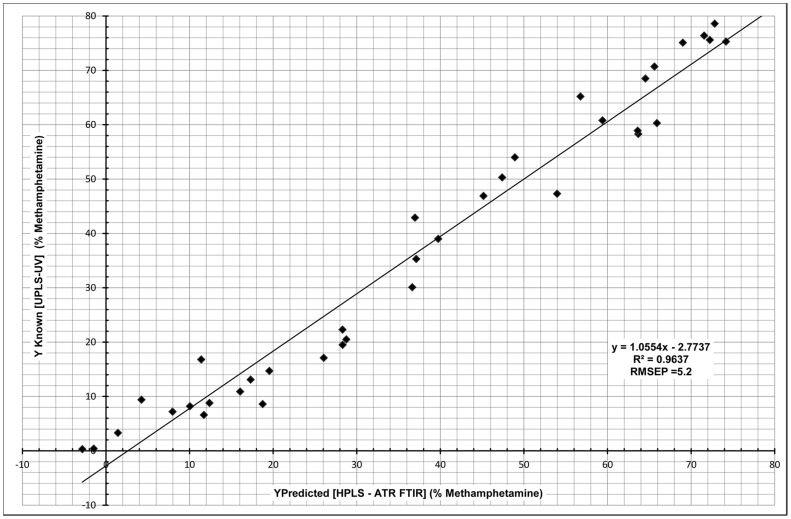
Methamphetamine HPLS Regression.

The HPLS model training set contained objects with MA % from 0.01% to 75.8%. It had 2 significant components explaining 98.3% of the variance in the X space, 96.7% of variance in the Y space and a cumulative Q^2^ = 96.5%. The model showed a very close cross validation, with the difference between cumulative R^2^Y and the cumulative Q^2^ value  = 0.2%. Response permutation gave values of MA intercepts: R^2^Y = −0.0487 and Q^2^ = −0.244. In addition, the training set had a RMSEE  = 4.7; the test set had RMSEP  = 5.2 and R^2^ = 0.9637. (See figure 4).

The significance of variable importance was greater than 0.9 for all variables with several of them ≥1.0 and the range in value of variable importance only 0.164, indicating similar importance; and when compared with the literature values outlined by Sun [9] with 0.5 the level of significance, these are all very significant. All these factors combined indicate this is a very valid model. The highest values of variable importance belonged to those variables whose vectors are coincident with PC1 and the % concentration of MA, see figures 1, 2. This indicates that the greatest variation in the HPCA analysis is related to the concentration of MA, as expected. These variables were variable 61, comprising 98.2% of variation of the 2400–2650 cm^−1^ region; and variable 71, comprising 97.9% of variation of the 2650–2900 cm^−1^ region. Note: In the previous model, the highest variable importance values were associated with the peaks that dominate these two regions, see figure 1, showing that these two peaks are very important in the quantification of methamphetamine regardless of model used.

The HPLS test set contained values from 0.3% up to 78.6%, and it included all of the cutting agents, including milk powder, which was excluded from the MA Principal Peaks PLS as all samples containing this cutting agent had MA concentration below the lower limit of quantification. While the R^2^ values (96.37% vs. 97.79%) and the RMSEP values (5.2 vs. 3.8) were not as good in the HPLS model as in the MA Principal Peak PLS analysis, this method was a substantial improvement (of more than one order of magnitude) in the detection of very low concentration samples from 7% in the MA Principal Peaks PLS to 0.3% in this model.

### 3) Method Efficacy

Both the MA Principal Peaks PLS model and the HPLS model are appropriate for quantifying methamphetamine. The Principal Peak PLS model gave an excellent RMSEP (3.8) and R^2^ (0.9779) values, with LLOQ (7% MA). The HPLS model however, had LLOQ (0.3% MA), R^2^ (0.9637) and RMSEP (5.2) values.

There is an excellent correlation between the currently used UPLC-UV method and this new proposed FTIR/ATR method. This is even more impressive as the current method uses chromatography to separate the mixture and the analysis targets only methamphetamine, whereas, this new method analyses the “dirty” mixture which contains unknown components without separating them and with little pre-preparation required. If the increased speed and decreased cost of analysis is also considered, this proposed FTIR/ATR method would be a rapid and robust additional/alternative method to analyse methamphetamine or other drugs.

While every effort was made to use the most comprehensive training set possible, the results were limited to samples with the available cutting agents (i.e. MSM, which was by far the most common, milk powder and various artificial sweeteners). One of the cutting agents that was in the training set (powdered milk/infant formula) was found to be, as noted above, an outlier in the principal peaks PLS analysis as all of the samples available had MA in concentrations below the limit of quantification. However in the HPLS, the final model contained samples with all the available cutting agents.

It should be remembered that other methamphetamine samples with different cutting agents may impact the accuracy of the results. [3] In that case periodically updating the method, with samples of methamphetamine containing a representative range of concentrations of the new cutting agent, may be required.

**Figure 5:**
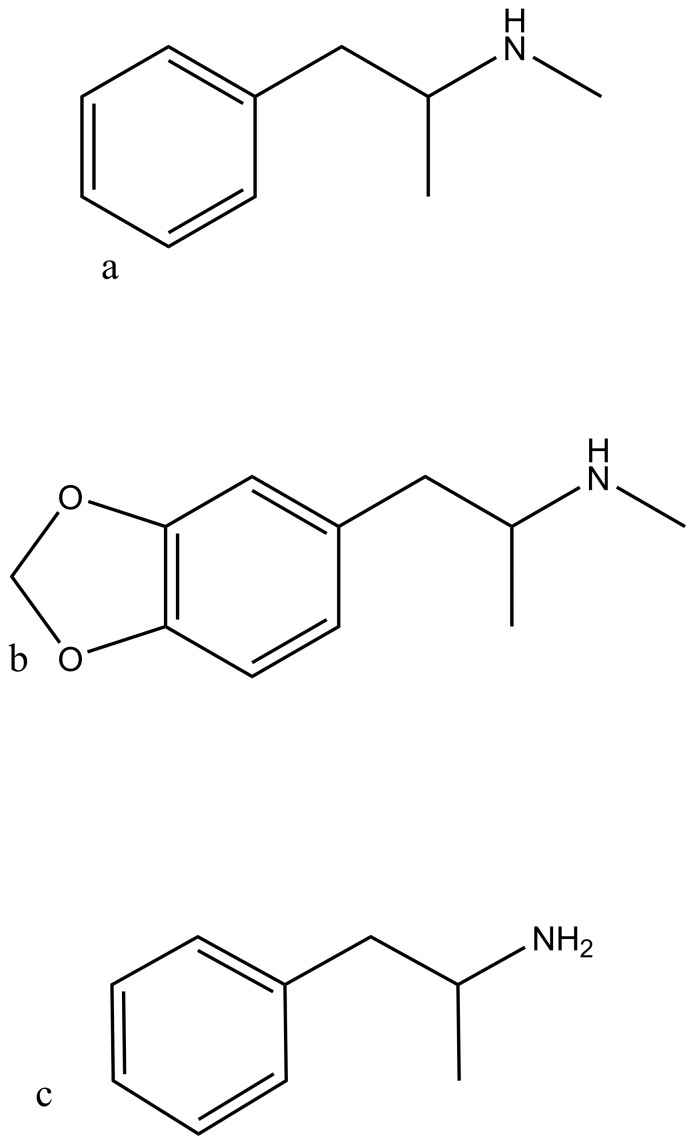
Structures of the most commonly encountered ATS, a) Methamphetamine, b) Ecstasy (MDMA: 3, 4 methylenedioxymethamphetamine), c) Amphetamine.

While these models showed that MA can be distinguished from the cutting agents, they may not distinguish between methamphetamine and other members of the ATS group, especially amphetamine and Ecstasy (MDMA: 3, 4 methylenedioxymethamphetamine), which have very similar chemical structure to methamphetamine, compare figure 5 parts a, b and c. Therefore most of their principal FTIR peaks would be at similar locations to those of methamphetamine.

As a consequence, the identification of methamphetamine, and its distinction from MDMA or amphetamine would require further research, developing a new model including samples of these other two illicit drugs in a range of representative concentrations. Or at the very minimum, some samples of mixtures containing these drugs would need to be analysed to verify that the models are valid. An HPCA model would be more likely to distinguish between methamphetamine and the 2 other compounds, as it includes more of the spectral data. Different peaks in the C-H stretching region would be expected in the MDMA and amphetamine spectrums. There would be C-O stretching peaks in the MDMA Spectrum, which may be used to discriminate it from the other compounds. Amphetamine, as a primary amine, would potentially also have different C-N and N-H peaks than the other two particularly in the NH stretching region (3300–3500 cm^−1^) where methamphetamine has weak or missing peaks, see figure 1.

These methods could be used as a rapid alternative to the current UPLC-UV method to: assist in undercover police operations, allow police to assess what charges should be laid and help streamline the committal process. With an appropriate, portable ATR-FTIR instrument, these combined methods could also be used for rapid in-field analysis. This may be useful, for example, in regional centres. The technique used in conjunction with a method of drug identification, e.g. a method developed using FTIR as suggested above or Ion Mobility Spectrometry– which is widely used in airport security, would allow for rapid indicative tests, assisting in ongoing investigations, and determination of charges. This technique would be particularly useful, as, limited training is required and no particular analytical skills are needed when the software is automated.

### Conclusion

This paper has developed new robust methods using ATR-FTIR and PLS as rapid and inexpensive alternatives to the current UPLC-UV method of MA quantification. When used in conjunction with a suitable method to identify the sample as MA, the methods will lead to significant savings in both time and public expenditure in the prosecution of illicit drug offenders.

## References

[1] United Nations Office on Drugs and Crime (2012) World Drug Report.

[2] Queensland Government (2010) Civil and Criminal Jurisdiction Reform and Modernisation Amendment Act.

[3] Goh CY, van Bronswijk W, Priddis C (2008) Rapid Nondestructive On-Site Screening of Methamphetamine Seizures by Attenuated Total Reflection Fourier Transform Infrared Spectroscopy. Applied Spectroscopy 62: 640–648. 18559151 10.1366/000370208784658002

[4] Bukowski EJ, Monti JA (2007) FTIR-ATR Spectroscopy for Identification of Illicit Drugs Seized from Clandestine Laboratories. American Laboratory 39: 16–19.

[5] Baran O (2005) Determination of Narcotic and Psychotropic Substances by Using Infrared Spectroscopy: Middle East Technical University.

[6] López-Sánchez M, Domínguez-Vida A, Ayora-Cañada MJ, Molina-Díaz A (2008) Assessment of dentifrice adulteration with diethylene glycol by means of ATR-FTIR spectroscopy and Chemometrics. Analytica Chimica Acta 620: 113–119. 18558131 10.1016/j.aca.2008.05.032

[7] Silvaa FEB, Ferrãob MF, Parisottoc G, Müllerc EI, Flores EMM (2008) Simultaneous determination of sulphamethoxazole and trimethoprim in powder mixtures by attenuated total reflection-Fourier transform infrared and multivariate calibration. Journal of Pharmaceutical and Biomedical Analysis 49: 5647–5657. 10.1016/j.jpba.2008.12.011 19179030

[8] Janné K, Pettersen J, Lindberg NO, Lundstedt T (2001) Hierarchical principal component analysis (PCA) and projection to latent structure (PLS) technique on spectroscopic data as a data pretreatment for calibration. Journal of Chemometrics 15 203–213.

[9] Sun H (2004) A Universal Molecular Descriptor System for Prediction of LogP, LogS, LogBB, and Absorption. J Chem Info and Comput Sci 44: 748–757. 10.1021/ci030304f 15032557

[10] Eriksson L, Hagberg P, Johansson E, Rännar S, Whelehan O, et al (2001) Multivariate process monitoring of a newsprint mill. Application to modelling and predicting COD load resulting from de-inking of recycled paper. Journal of Chemometrics 15: 337–352.

